# Correlation between Serum Levels of Nitric Oxide and Adropin and Erectile Dysfunction in Males with Nonalcoholic Fatty Liver Disease: An Observational Study

**DOI:** 10.1007/s43032-024-01537-4

**Published:** 2024-04-30

**Authors:** Ahmed Ragab, Ali M Abdel Fattah, Ahmed Reda Sayed, Sameh Fayek GamalEl Din, Shrouk Matrawy Mahmoud Hassan, Aya Yaseen Mohamed Mohamed, Mostafa Ahmed Hamed

**Affiliations:** 1 Department of Andrology, Sexology and STDs, Faculty of Medicine, Beni-Suef University, BeniSuef, Egypt; 2 Department of Gastroenterology, Hepatology and Endemic Medicine, Faculty of Medicine, Beni-Suef University, BeniSuef, Egypt; 3 Department of Medical Biochemistry and Molecular Biology, Faculty of Medicine, Beni-Suef University, BeniSuef, Egypt; 4 Department of Andrology, Sexology and STDs, KasrAlainy Faculty of Medicine, Cairo University, Al-Saray Street, El Manial, Cairo, 11956 Egypt; 5 Beni-Suef Mental Health Hospital, BeniSuef, Egypt

**Keywords:** Adropin, Nitric oxide, Creatinine, GAD-7, NAFLD

## Abstract

The current study aimed to evaluate the serum levels of nitric oxide (NO) and adropin in males with non-alcoholic fatty liver disease (NAFLD) induced erectile dysfunction (ED) and NAFLD patients without ED and controls. The current study selected 165 participants from the hepatology department from November 2021 to November 2022. The patients were either suffering from NAFLD with normal liver functions or non-alcoholic steatohepatitis with abnormal liver functions. They were diagnosed by abdominal ultrasonography. Participants were evaluated using the validated Arabic version of the International Index of Erectile Function (ArIIEF-5), the Arabic form of the Generalized Anxiety Disorder-7 (GAD-7) questionnaire and the Patient Health Questionnaire-9 (PHQ-9). Noteworthy, there were significant positive correlations between ArIIEF-5 score, NO, adropin and total testosterone (r = 0.380, p = 0.001; r = 0.507, p =  < 0.001; r = 0.246, p = 0.038, respectively). Meanwhile, there were significant negative correlations between ArIIEF-5 score, creatinine, duration of the disease and scores of GAD-7 and PHQ-9 (r = -0.656, p =  < 0.001; r = -0.368, p = 0.002; r = -0.663, p =  < 0.001; r = -0.248, p = 0.037, respectively). Finally, a linear regression analysis revealed that GAD-7, creatinine, and adropin were the only strong independent predictors of ArIIEF-5, as the 95% confidence interval in the form of upper and lower bounds was -0.349, -0.843, p < 0.001, -6.507, -18.402, p < 0.001, 0.476, 0.117, and p 0.002, respectively. Impaired NO and adropin levels play a potential role in the development of ED in patients with NAFLD.

## Introduction

Erectile dysfunction (ED) is one of the most common sexual dysfunctions in men [1, 2]. It is regarded as the inability to initiate and/or maintain an erection adequate for intercourse [3]. ED is thought to be quite common among those with liver disease [4, 5]. Non-alcoholic fatty liver disease (NAFLD) is defined as the accumulation of intrahepatic fat of at least 5% of liver weight in absence of a secondary contributing factor or therapeutic drugs [6, 7]. It is the most common chronic liver disease in developed countries, affecting more than 30% of the global population [5, 8, 10]. NAFLD is regarded as the hepatic manifestation of metabolic syndrome (MetS), which is associated with central obesity, glucose intolerance, hyperinsulinemia, dyslipidemia and/or hypertension [11]. Corona et al. (2023) stated that ED associated with MetS was multifactorial with the necessity of treating such risk factors individually to manage ED [12]. Consistently, an observational study demonstrated an association between ED and celiac disease [13]. Also, the same study revealed that early age at diagnosis together with high body mass index were the main determinants for ED in this group of patients [13]. Remarkably, other conditions could cause ED including obstructive sleep apnea that was associated with ED in a study conducted by Cantone et al. (2022) [14]. Vasculogenic ED is the most frequent reason for ED in patients with NAFLD [5]. Nitric oxide (NO) is released from nerves in the corpora cavernosa on sexual stimulation and is one of the important molecules controlling the process of erection [15]. The NO pathway has other vital roles in the human body [16, 17]. Shared factors for reduced NO bioavailability in the human body are sedentary lifestyles, ageing, poor nutrition, oxidative stress, cardiovascular diseases, nephropathies and antidepressant drugs [16, 18–20].

Human data demonstrated that patients with NAFLD had marked endothelial NO synthase (eNOS) dysfunction and animal data demonstrated that eNOS deficiency exacerbated the early stages of NAFLD [21, 22]. Adropin was first identified in the liver and brain tissues of rats [21]. Its secretion is mediated by dietary macro-nutrients [23]. Recently, serum adropin was observed to decrease in patients with NAFLD [24]. Although several studies [5, 25, 26] highlighted the association between NAFLD and ED, no study before addressed the relationship between patients with NAFLD induced ED and serum levels of NO and adropin. Furthermore, exploratory studies were needed to unravel the underlying pathophysiology responsible for this potential association [26]. Thus, we aimed to evaluate the serum levels of NO and adropin in males with NAFLD induced ED and NAFLD patients without ED and controls. Moreover, we aimed to study the relationship between serum NO and adropin levels and the degree of ED in males with NAFLD.

## Methods

The current observational study selected 165 participants from the hepatology department of Beni-Suef University Hospital from November 2021 to November 2022. Males with NAFLD were allocated into either NAFLD induced ED (group I; n = 41) or NAFLD with preserved erectile function (group II; n = 30). Moreover, 94 controls (group III) were recruited as well. Eligible participants were asked to sign an informed consent prior to enrolment in this study, according to the regulations mandated by the Research Ethical Committee (REC) of Beni-Suef Faculty of Medicine that conform to the Helsinki Declaration (2013) (Approval Number: FMBSUREC/07092021–01112021) [27].

### Inclusion Criteria for Patients

All patients should be in a steady sexual relationship during the last six months. Patients were men aged more than 20 years with the following diagnostic criteria of NAFLD: signs of fatty liver by abdominal US through detecting increased echogenicity of the liver versus the kidneys and the spleen [28] plus overweight or obesity (BMI > 25) or two of the following criteria of MetS: increased waist circumference: > 102 cm; blood pressure > 130/ 85; plasma triglyceride > 150; plasma HDL cholesterol < 40 and pre-diabetic status in which fasting blood glucose level was 100 to 125 mg /dl or HbA1c 5.7% to 6.4% or 2 h post-prandial blood glucose levels 140 to 199 [29].

### Inclusion Criteria for Controls

Controls were age and BMI-matched healthy volunteers. They were companions of the patients who attended the hepatology department seeking for diagnosis and treatment of their condition.

### Exclusion Criteria

Patients with liver cirrhosis, diabetes, psychogenic ED or patients receiving drugs associated with ED were excluded. Also, end-stage renal disease, neurological diseases, COPD, coronary artery disease, endocrine disorders, hepatic fat accumulation secondary to excessive alcoholism (intake of more than 30 g/day), long-term use of steatogenic drugs, viral hepatitis and hereditary hemochromatosis were also excluded.

All participants were subjected to the following:

Past and medical histories were obtained from all participants. Evaluation of erectile function was done using the validated Arabic version of the international index of erectile function (ArIIEF-5) [30]. Evaluation of anxiety manifestations was done using the validated Arabic form of the Generalized Anxiety Disorder-7 (GAD-7) questionnaire [31, 32].Evaluation of depression manifestations was done using the Patient Health Questionnaire-9 (PHQ-9) [32, 33]. General and genital examinations were done. Fasting plasma glucose, glycosylated hemoglobin, serum lipid profile and hepatic function panel were all obtained via a blood sample that was withdrawn from the participants. A fasting sample before 11 AM was taken for assessment of total testosterone serum levels (ng/dl). NO levels were measured in venous blood samples using a colorimetric assay kit from Elabscience Biotechnology Co. (Wuhan, China). The concentration of NO was determined based on the optical density value at 550 nm.The assay kit had low coefficients of variation for both intra-assay and inter-assay measurements, with values of 2.4% and 3.7%, respectively. The NO assay range was 0.16–100 μmol/l with a sensitivity of 0.16 μmol/l. Additionally, serum adropin levels were measured using an enzyme-linked immunosorbent assay (ELISA) kit from the same manufacturer. The coefficients of variation for the adropin assay were less than 10%. The detection range for adropin was 12.50–800 pg/mL and the assay exhibited a sensitivity of 7.50 pg/mL. Venous blood samples were collected after a 12-h overnight fasting period. The patients were either suffering from NAFLD with normal liver functions or non-alcoholic steatohepatitis with abnormal liver functions (NASH). Liver biopsies were not used to differentiate between both types of NAFLD [34, 35]. Differentiation between both types depended on clinical signs of hepatitis and liver functions [34, 35].

### Sample Size Determination

The prevalence of NAFLD is generally 25%, in line with the findings of a prior study conducted by Young and colleagues (2020) [36]. Supposing a 95% confidence interval (CI) with an error bound of 0.05, at least a sample size of 100 participants should be recruited based on the Epi Info 7 main menu. The number of participants was maximized to 165 to intensify the findings of this study.

### Statistical Analysis

Data analysis was completed using the Statistical Package for Social Sciences (SPSS) (version 26.0, IBM, Chicago). Qualitative and quantitative data were expressed using frequency (%) and mean + S.D., respectively. Variables were compared using the t test, one-way ANOVA, and X2 test. The Pearson coefficient (r) was utilized to evaluate correlations.

## Results

Table 1 shows the socio-demographic characteristics of the participants. Nineteen cases suffered from moderate ED (46.3%), whereas the rest of the cases were equally split into mild ED (26.8%) and severe ED (26.8%). Furthermore, 17 cases suffered from minimal depression (41.5%) and 17 cases suffered from mild depression (41.5%). whereas 4 cases and 3 cases suffered from severe depression (9.8%) and moderate depression (7.3%), respectively. Additionally, 19 cases and 14 cases were mildly anxious (46.3%) and moderately anxious (34.1%), respectively. A pair-wise comparison between NAFLD induced ED and NAFLD without ED and controls was shown in Table 2. Noteworthy, there were significant positive correlations between the ArIIEF-5 score, NO, adropin and total testosterone (Table 3).

**Table 1:** Socio-demographic characteristics of the participants

	NAFLD with ED (n = 41)		NAFLD without ED (n = 30)		Control (n = 94)		P value
	Mean	SD	Mean	SD	Mean	SD	
Age (years)	40.76	6.99	38.43	5.47	38.47	5.19	0.215
Weight(Kg)	92.67	13.02	95.47	13.34	93.74	9.44	0.648
Height (m)	1.72	0.07	1.74	0.07	1.74	0.07	0.417
BMI (kg/m^2^)	26.91	3.12	27.26	3.63	26.83	2.26	0.866
Waist (cm)	112.83	8.68	113.43	8.72	103.95	5.92	< 0.001
SBP (mmHg)	121.80	9.58	125.00	14.08	118.16	6.28	0.100
DBP (mmHg)	79.63	6.74	81.50	8.42	77.37	5.62	0.147
ALT (U/L)	40.19	17.41	44.03	26.43	25.42	4.46	0.005
AST (U/L)	33.52	13.29	37.87	16.83	24.74	5.59	0.005
Creatinine(mg/dl)	1.02	0.14	0.82	0.20	0.77	0.21	< 0.001
Serum albumin(g/dL)	4.48	0.46	4.36	0.44	4.45	0.45	0.520
INR	1.00	0.02	1.01	0.03	1.01	0.02	0.689
PT (seconds)	12.12	0.46	12.01	0.05	12.44	0.83	0.013
Total Bilirubin (mg/dL)	0.80	0.25	0.76	0.22	0.73	0.22	0.572
FPG (mg/dL)	93.80	12.68	90.53	14.65	81.00	8.30	0.002
HbA1c (%)	5.49	0.46	5.42	0.45	5.12	0.26	0.007
PPG (mg/dL)	123.73	21.68	120.63	25.90	114.21	10.82	0.286
Total cholesterol (mg/dl)^)^	192.10	44.26	173.37	41.81	163.37	24.15	0.023
Triglycerides (mg/dl)	167.29	65.44	150.27	91.06	118.89	23.10	0.048
HDL (mg/dl)	44.22	8.00	44.47	5.82	45.95	5.20	0.647
LDL (mg/dl)	101.36	37.86	89.17	39.08	70.00	12.37	0.006
ArIIEF-5 score	10.95	4.26	22.73	0.74	23.74	0.65	< 0.001
Adropin (pg/mL)	16.96	4.54	21.77	6.45	25.18	6.50	< 0.001
NO (μmol/l)	13.38	3.00	16.04	5.25	19.24	5.03	< 0.001
TT (ng/ml)	2.51	1.01	2.59	1.09	3.22	0.97	0.018
Duration of disease(years)	6.90	3.43	4.57	3.05	.-	-	0.005
PHQ-9 score	5.83	5.12	3.27	3.06	1.79	1.08	0.006
GAD-7 score	10.20	3.71	4.23	3.29	1.95	1.18	< 0.001

P value was calculated using an unpaired *t*-test or ANOVA

Abbreviations: ALT-alanine aminotransferase; AST-aspartate aminotransferase;BMI-body mass index; DBP-diastolic blood pressure; FPG-fasting plasma glucose; GAD-7-generalized anxiety disorder-7; HbA1c- glycosylated hemoglobin; HDL-high-density lipoproteins; ArIIEF-5- the validated Arabic version of the international index of erectile function-5; INR-international normalized ratio; LDL-low density lipoproteins; NO-nitric oxide; PHQ-9- patient health questionnaire-9; PPG postprandial plasma glucose; PT- prothrombin time; SBP-systolic blood pressure; TT-total testosterone

**Table 2:** Pairwise comparisons between different groups

	NAFLD with ED VS NAFLD without ED	NAFLD with ED VS Controls	NAFLD without ED VS Controls
Waist (cm)	1.000	0.001	< 0.001
ALT	1.000	0.022	0.005
AST	0.550	0.064	0.004
Creatinine	< 0.001	< 0.001	1.000
PT	1.000	0.069	0.012
FBS	0.853	0.001	0.035
HbA1c	1.000	0.006	0.049
TC	0.164	0.034	1.000
TG	0.934	0.042	0.383
LDL	0.440	0.005	0.187
ArIIEF-5 score	< 0.001	< 0.001	0.113
Adropin	0.003	< 0.001	0.238
NO	0.036	< 0.001	0.076
TT	1.000	0.017	0.078
PHQ-9 score	0.087	0.003	0.319
GAD-7 score	< 0.001	< 0.001	0.082

Multiple comparisons post hoc test was used for statistical analysis

Abbreviations: ALT-alanine aminotransferase; AST-aspartate aminotransferase; FPG-fasting plasma glucose; GAD-7-generalized anxiety disorder-7; HbA1c- glycosylated hemoglobin; ArIIEF-5- the validated Arabic version of the international index of erectile function-5; LDL-low density lipoproteins; NO-nitric oxide; PHQ-9- patient health questionnaire-9; PPG postprandial plasma glucose; PT- prothrombin time; SBP-systolic blood pressure; TC–total cholesterol; TG-triglycerides; TT-total testosterone

**Table 3:** Correlations between Ar IIEF-5 and other parameters in NAFLD patients

	ArIIEF-5 score (n = 71)	
	r	P value
Age (years)	-0.226	0.058
Serum adropin (pg/mL)	0.507	< 0.001
Serum NO (μmol/l)	0.380	0.001
Serum TT (ng/ml)	0.246	0.038
Weight (kg)	-0.014	0.908
Height (m)	0.077	0.522
BMI (kg/m^2^)	-0.055	0.647
Waist (cm)	-0.038	0.751
Duration Of Disease (years)	-0.368	0.002
PHQ9 score	-0.248	0.037
GAD7 score	-0.663	< 0.001
SBP (mmHg)	0.188	0.117
DBP (mmHg)	0.211	0.078
ALT (U/L)	0.072	0.549
AST(U/L)	0.118	0.326
Creatinine (mg/dl)	-0.656	< 0.001
Albumin (mg/dl)	-0.168	0.161
INR	0.086	0.474
PT (seconds)	-0.166	0.165
Bilirubin (mg/dl)	-0.019	0.873
FPS (mg/dl)	-0.059	0.625
HbA1c (%)	-0.076	0.528
PPG (mg/dl)	-0.026	0.827
TC (mg/dl)	-0.117	0.332
TG (mg/dl)	-0.138	0.251
HDL (mg/dl)	-0.018	0.879
LDL (mg/dl)	-0.023	0.848

Variables were correlated using Pearson correlation

Abbreviations: ALT-alanine aminotransferase; AST-aspartate aminotransferase; BMI-body mass index; DBP-diastolic blood pressure; FPG-fasting plasma glucose; GAD-7-generalized anxiety disorder-7; HbA1c- glycosylated hemoglobin; HDL-high-density lipoproteins; ArIIEF-5 = the validated Arabic version of the international index of erectile function-5; LDL-low density lipoproteins; NO-nitric oxide; PHQ-9- patient health questionnaire-9; PPG postprandial plasma glucose; PT- prothrombin time; SBP-systolic blood pressure; TC–total cholesterol; TG-triglycerides; TT-total testosterone

**Fig. 1:**
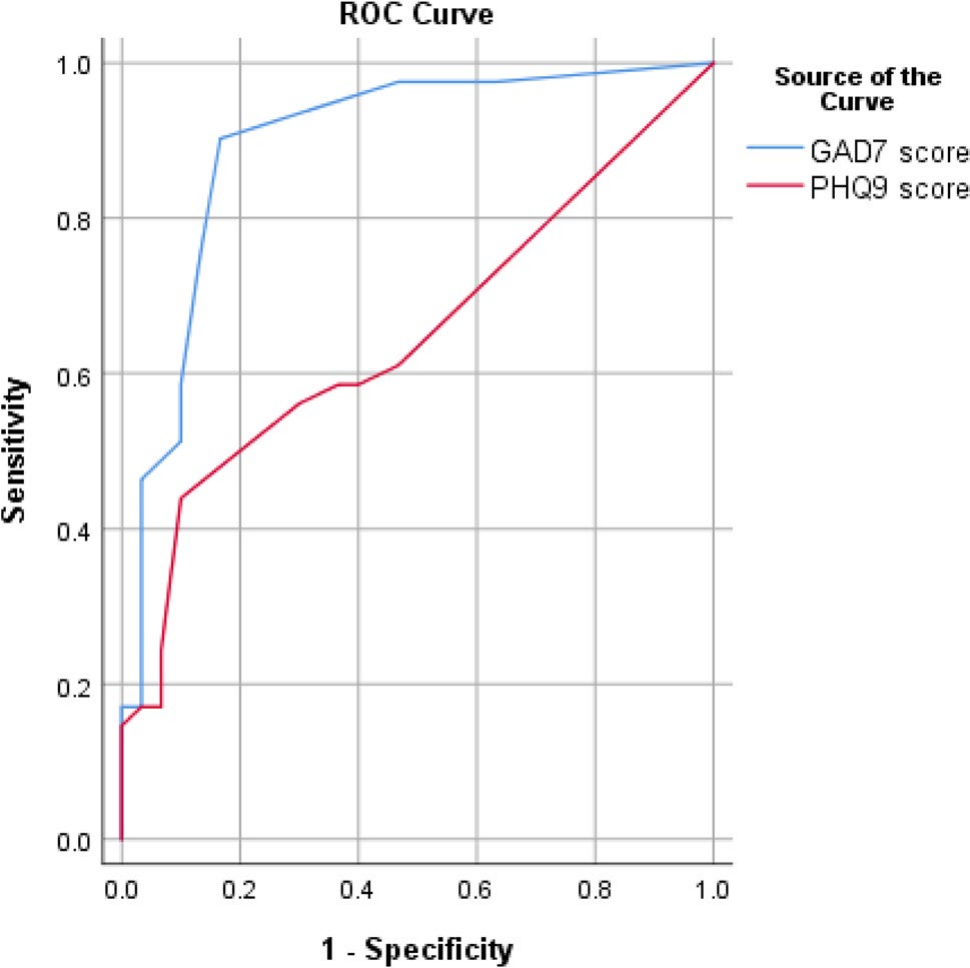
ROC curve for detection of erectile dysfunction in fatty liver patients using GAD7 and PHQ9

**Fig. 2:**
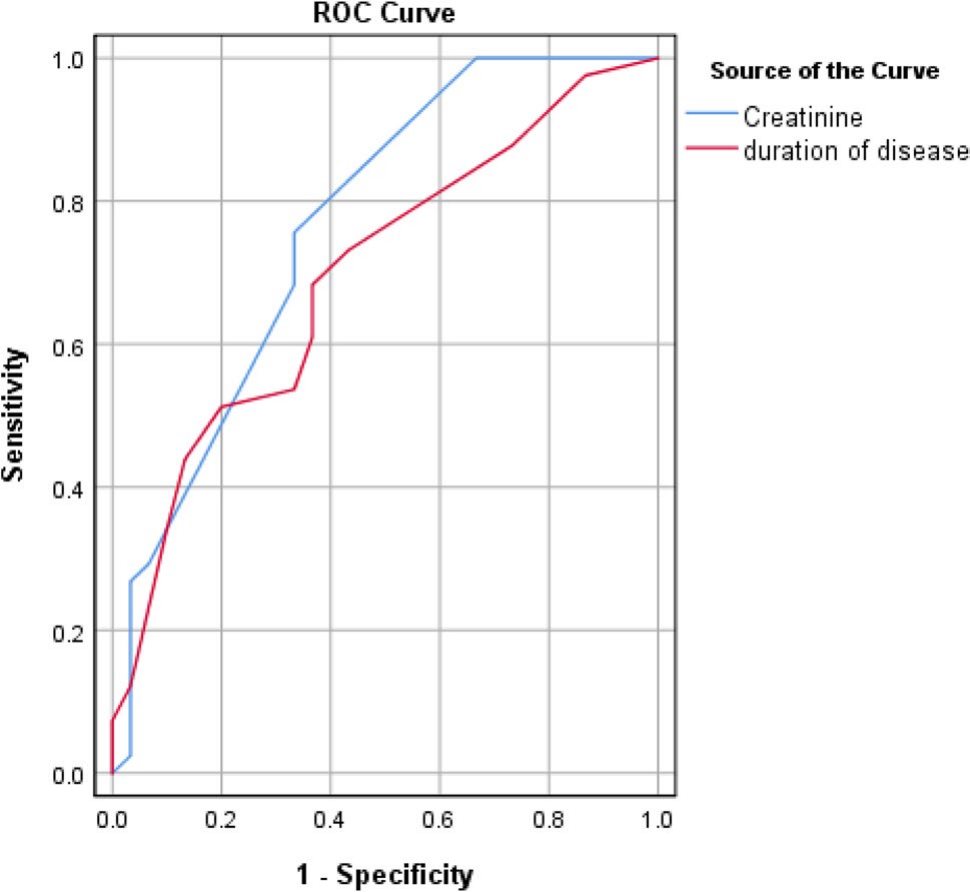
ROC curve for detection of erectile dysfunction in fatty liver patients using significant parameters

**Fig. 3:**
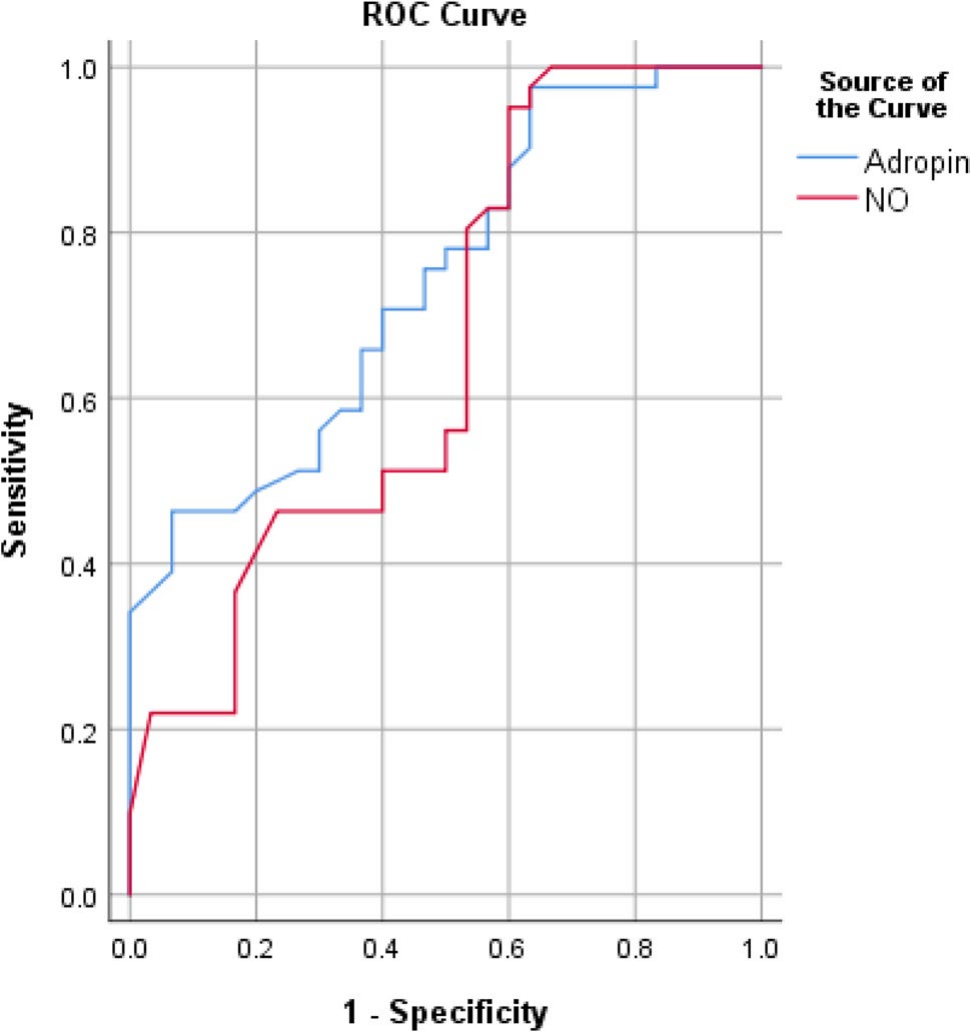
ROC curve for detection of erectile dysfunction in fatty liver patients using Adropin and NO

Meanwhile, there were significant negative correlations between the ArIIEF-5 score, creatinine, duration of the disease and scores of GAD-7 and PHQ-9 (Table 3). Receiver operating characteristic (ROC) curve for predicting ED in NAFLD patients using scores of GAD-7 revealed cutoff value and sensitivity and specificity were 6.5, 90.2%, and 83.3%, respectively (Fig. 1). Meanwhile, ROC curve for predicting ED in NAFLD patients using scores of PHQ-9 revealed cutoff value and sensitivity and specificity were 5.5, 56.1%, and 70%, respectively (Fig. 1). Thus, GAD-7 score was a strong predictor of ED. Furthermore, ROC curve revealed that cutoff value and sensitivity and specificity for predicting ED in NAFLD patients using the duration of the disease were 4.5, 68.3%, and 63.3%, respectively (Fig. 2). Meanwhile, ROC curve demonstrated that cutoff value and sensitivity and specificity of creatinine for predicting ED in NAFLD patients were 0.925, 75.6%, and 66.7%, respectively (Fig. 2). Finally, ROC curve demonstrated that cutoff values and sensitivity and specificity of adropin and NO for predicting ED in NAFLD patients were 17.95 ng/ml, 70.7% and 60%; 12.55, 51.2% and 60%, respectively (Fig. 3). Finally, a linear regression analysis revealed that GAD-7, creatinine and adropin were the only strong independent predictors of the ArIIEF-5 score as the 95% confidence interval (CI) in the form of upper and lower bounds were -0.349, -0.843, p < 0.001, -6.507, -18.402, p < 0.001, 0.476, 0.117, and p 0.002, respectively (Table 4).

**Table 4:** Multivariate linear regression analysis for detection of erectile dysfunction using ArIIEF-5 score after adjustment of different parameters

Model		Unstandardized Coefficients		Standardized Coefficients	t	P value	95.0% Confidence Interval for B	
		B	Std. Error	Beta			Lower Bound	Upper Bound
ArIIEF-5 score	(Constant)	26.525	3.519		7.538	< 0.001	19.501	33.549
	GAD7 score	-0.596	0.124	-0.409	-4.814	< 0.001	-0.843	-0.349
	Creatinine	-12.454	2.980	-0.363	-4.180	< 0.001	-18.402	-6.507
	Serum Adropin	0.297	0.090	0.261	3.300	0.002	0.117	0.476

Abbreviations: GAD-7-generalized anxiety disorder-7; ArIIEF-5- the validated Arabic version of the international index of erectile function-5

## Discussion

The current observational study demonstrated that there were significant positive correlations between the ArIIEF-5 score, NO, adropin and total testosterone. Meanwhile, there were significant negative correlations between the ArIIEF-5 score, creatinine, duration of the disease, and scores of GAD-7 and PHQ-9.

Also, a linear regression analysis revealed that GAD-7, creatinine, and adropin were the only strong independent predictors of ArIIEF-5. Endothelium-produced NO plays an important role in maintaining good erectile function [37]. Thus, decreased NO availability led to ED, which could be seen as similar to our finding [37]. Furthermore, decreased NO bioactivity has been depicted as the most important pathological mechanism in ED [38]. An animal study revealed that adropin contributed to NO bioavailability as well as impacting inducible NOS synthesis [39]. Consistently, Kutlu et al. (2019) showed decreased adropin in cases with NAFLD [24]. Additionally, Topuz et al. (2013) postulated that adropin level could be used as a marker to quantify endothelial dysfunction [40]. Thus, it was concluded that adropin had a detrimental effect on erectile function which was also in line with our finding. Testosterone has a direct regulatory effect on insulin secretion in men [41]. Thus, hyperinsulinemia is associated with low testosterone and an increased body mass index [41]. Hyperinsulinemia is associated with increased hepatic lipogenesis, adipose tissue lipolysis and efflux of free fatty acids to the liver with subsequent development of steatosis and progression to NAFLD [42]. Consistently, the chronic low-grade inflammatory state resulting from increased secretion of hepatic inflammatory cytokines such as tumour necrosis factor-α and interleukin-6 might directly suppress luteinizing hormone secretion from the pituitary gland which decreased testosterone secretion from Leydig cells [43]. Similarly, our study demonstrated that NAFLD was associated with low total testosterone that led to ED as a result of this deficiency which could be seen in line with a study conducted by Crocetto et al. (2022) [44]. It is well agreed that decreased testosterone is associated with impaired erectile function which can be diagnosed by the CATCH checklist [45].

In the same context, Ragab et al. (2023) revealed an association between testosterone deficiency and ED [46]. Remarkably, there are similar traditional risk factors for chronic kidney disease and NAFLD, including hypertension, obesity, dyslipidemia, and insulin resistance [47]. Thus, it is difficult to determine a causal relationship with NAFLD adjusting for “hepato-renal” and “cardio-renal” features [47]. Hypothetically, increased caloric intake and fatty tissue induce an inflammatory cascade through the energy sensor 5´-AMP activated protein kinase, fetuin-A and adiponectin between fat cells in the liver and the kidney, leading to end-organ damage [48]. The renin-angiotensin system had been postulated as a player in the pathogenesis of NAFLD and chronic kidney disease (CKD) [46]. Henceforth, NAFLD was associated with renal dysfunction [50]. ED could be used as an early predictor of CKD, as there was a strong relationship between endothelial dysfunction and ED and the evolution of systemic diseases [51, 52]. Consistently, a recent Egyptian study revealed a significant association between IIEF-5 and creatinine [46]. Hasanain et al. (2017) found significant associations between ageing, obesity and hypertension and patients with NAFLD induced ED [53].This could be seen in line with our finding, as a significant association between duration of the disease and patients with NAFLD induced ED was demonstrated in the current study. Recently, Ragab et al. (2023) showed a significant correlation between the GAD-7 score and ED [46]. Similarly, an Indian randomized clinical trial showed that a multidisciplinary care for 12 months compared to the usual care resulted in statistically significant improvements in depressive symptoms and cardio-metabolic indices at 24 months [54]. Interestingly, the current study has shown that adropin, creatinine, and GAD-7 are the only significant predictors of ED in NAFLD patients.

These findings could be expected from the pivotal role of adropin in NO synthesis as well as the strong association between NAFLD and CKD that was extensively discussed in the current study. To wrap up the findings of the current study, adropin can be used as a marker to predict ED in NAFLD patients taking into consideration the relatively low cost of the kits for measuring NO and adropin in patients suffering from NAFLD. Additionally, it is critical to prioritize psychiatric care and closely monitor kidney function in patients with NAFLD. Admittedly, the small number of patients as well as being recruited from a single center could be seen as a major limitation. Also, the nature of the study being an observational one could be added as another limitation. However, to the best of our knowledge this study is one of the first to show a link between NAFLD induced ED and NO and adropin. Thus, we didn’t have enough previous studies to properly calculate the sample size. Also, after statistical revision to calculate the power analysis of the study regarding the primary outcome, it was around 99% which pushed us to refrain from adding it owing to the significant relationships revealed between NAFLD induced ED and NO and adropin in our small sample size. Finally, the degree of NAFLD was not mentioned in the current study, despite the fact that it could be graded by abdominal ultrasound into three grades or scores according to the degree of liver echogenicity [28]. However, we did not mention the grades of NAFLD by ultrasound in our research as there is no rationale to classify the patients according to the grades of NAFLD by ultrasonography as we had a small sample size, not allowing more sub-grouping of patients according to the grades of NAFLD. Thus, we thought it would be better to analyze and interpret the data of the total patients without sub-grouping according to their grades.

## Conclusion

Impaired NO and adropin levels play a potential role in the development of ED in patients with NAFLD. Consequently, patients suffering from NAFLD should be screened for NO and adropin to predict the occurrence of ED and manage accordingly. Also, it is critical to prioritize psychiatric care and closely monitor kidney function in patients with NAFLD. Future longitudinal cohort studies are recommended to assert these findings.

## Data Availability

The data that supports the funding of this study are not publicly available due to their containing information that could compromise the privacy of research participants. But the data are available from the corresponding author, S.F. (samehfayek@hotmail.com), upon reasonable request.

## Code Availability

Code availability Data were coded and entered using the statistical package for the Social Sciences (SPSS) version 26 (IBM Corp., Armonk, NY, USA).

## References

[1] Martin SA, Atlantis E, Lange K, et al, members of the Florey Adelaide Male Ageing Study. Predictors of sexual dysfunction incidence and remission in men. J Sex Med 2014; 11:1136–47. 10.1111/jsm.12483 24548342

[2] Gades NM, Jacobson DJ, McGree ME, et al. Longitudinal evaluation of sexual function in a male cohort: the Olmsted County Study of Urinary Symptoms and Health Status among Men. J Sex Med. 2009;6:455. 10.1111/j.1743-6109.2009.01374.x PMC2862565 19570040

[3] NIH Consensus Conference. Impotence. NIH Consensus Development Panel on Impotence. JAMA.1993;270:83–90. 8510302

[4] Durazzo M, Premoli A, Di Bisceglie C, Bo S, Ghigo E, Manieri C. Male sexual disturbances in liver diseases: what do we know? J Endocrinol Invest. 2010;33(7):501–5. 20671409 10.1007/BF03346632

[5] Tarik Kani H, Emre Sener T, Emre Aykut U, OzerDemirtas C, Keklikkiran C, Ergenc I, Fatih Demirci A, Kamil Cam H, Celikel C, Akbal C, Duman D. Causes of erectile dysfunction in non-alcoholic fatty liver disease. Hepatol Forum. 2021;2(2):60–3. 35783901 10.14744/hf.2021.2021.0012 PMC9138920

[6] Shiha G, Korenjak M, Eskridge W, Casanovas T, Velez-Moller P, Högström S, Richardson B, Munoz C, Sigurðardóttir S, Coulibaly A, Milan M, Bautista F, Leung NWY, Mooney V, Obekpa S, Bech E, Polavarapu N, Hamed AE, Radiani T, Purwanto E, Bright B, Ali M, Dovia CK, McColaugh L, Koulla Y, Dufour JF, Soliman R, Eslam M. Redefining fatty liver disease: an international patient perspective. Lancet Gastroenterol Hepatol. 2021;6(1):73–9. 33031758 10.1016/S2468-1253(20)30294-6

[7] Idilman IS, Ozdeniz I, Karcaaltincaba M. Hepatic Steatosis: Etiology, Patterns, and Quantification. Semin Ultrasound CT MR. 2016;37(6):501–10. 27986169 10.1053/j.sult.2016.08.003

[8] Williams CD, Stengel J, Asike MI, Torres DM, Shaw J, Contreras M, et al. Prevalence of nonalcoholic fatty liver disease and nonalcoholic steatohepatitis among a largely middle-aged population utilizing ultrasound and liver biopsy: a prospective study. Gastroenterology. 2011;140(1):124–31. 20858492 10.1053/j.gastro.2010.09.038

[9] Ahmed A, Wong RJ, Harrison SA. Nonalcoholicfattyliverdisease review: diagnosis, treatment, and outcomes. Clin Gastroenterol Hepatol. 2015;13(12):2062–70. 26226097 10.1016/j.cgh.2015.07.029

[10] Hawksworth DJ, Burnett AL. Nonalcoholic fatty liver disease, male sexual dysfunction, and infertility: common links, common problems. Sexual Med Rev. 2020;8(2):274–85. 10.1016/j.sxmr.2019.01.002 30898592

[11] Haas JT, Francque S, Staels B. Pathophysiology and mechanisms of nonalcoholic fatty liver disease. Annu Rev Physiol. 2016;78(1):181–205. 26667070 10.1146/annurev-physiol-021115-105331

[12] Corona DG, Vena W, Pizzocaro A, Rastrelli G, Sparano C, Sforza A, Vignozzi L, Maggi M. Metabolic syndrome and erectile dysfunction: a systematic review and meta-analysis study. J Endocrinol Invest. 2023;46(11):2195–211. 37515706 10.1007/s40618-023-02136-x

[13] Romano L, Pellegrino R, Sciorio C, Barone B, Gravina AG, Santonastaso A, Mucherino C, Astretto S, Napolitano L, Aveta A, Pandolfo SD, Loizzo D, Del Giudice F, Ferro M, Imbimbo C, Romano M, Crocetto F. Erectile and sexual dysfunction in male and female patients with celiac disease: A cross-sectional observational study. Andrology. 2022;10(5):910–918. 10.1111/andr.13186. PMID: 35419983; PMCID: PMC9324123. 10.1111/andr.13186 PMC9324123 35419983

[14] Cantone E, Massanova M, Crocetto F, Barone B, Esposito F, Arcaniolo D, Corlianò F, Romano L, Motta G, Celia A. The relationship between obstructive sleep apnoea and erectile dysfunction: An underdiagnosed link? A prospective cross-sectional study. Andrologia. 2022;54(9):e14504. 10.1111/and.14504. PMID: 35817418; PMCID: PMC9539465. 10.1111/and.14504 PMC9539465 35817418

[15] Ralph DJ. Normal erectile function. Clin Cornerstone. 2005;7(1):13–7. 16156419 10.1016/S1098-3597(05)80044-4

[16] Raddino R, Caretta G, Teli M, Bonadei I, Robba D, Zanini G, Madureri A, Nodari S, Dei CL. Nitric oxide and cardiovascular risk factors. Heart Int. 2007;3(1–2):1826186807003001–203. 10.4081/hi.2007.18 PMC3184682 21977271

[17] Ahluwalia A, Gladwin M, Coleman GD, Hord N, Howard G, Kim-Shapiro DB, Lajous M, Larsen FJ, Lefer DJ, McClure LA, Nolan BT. Dietary nitrate and the epidemiology of cardiovascular disease: report from a National Heart, Lung, and Blood Institute Workshop. J Am Heart Assoc. 2016;5(7):e003402. 27385425 10.1161/JAHA.116.003402 PMC5015377

[18] Luiking YC, Engelen MP, Deutz NE. Regulation of nitric oxide production in health and disease. Curr Opin Clin Nutr Metab Care. 2010;13(1):97. 19841582 10.1097/MCO.0b013e328332f99d PMC2953417

[19] Razny U, Kiec-Wilk B, Wator L, Polus A, Dyduch G, Solnica B, Malecki M, Tomaszewska R, Cooke JP, Dembinska-Kiec A. Increased nitric oxide availability attenuates high fat diet metabolic alterations and gene expression associated with insulin resistance. Cardiovasc Diabetol. 2011;10(1):1–4. 21781316 10.1186/1475-2840-10-68 PMC3212914

[20] Saroukhani S, Emami-Parsa M, Modabbernia A, Ashrafi M, Farokhnia M, Hajiaghaee R, Akhondzadeh S. Aspirin for treatment of lithium-associated sexual dysfunction in men: randomized double-blind placebo-controlled study. Bipolar Disord. 2013;15(6):650–6. 23924261 10.1111/bdi.12108

[21] Nozaki Y, Fujita K, Wada K, et al. Deficiency of eNOS exacerbates early-stage NAFLD pathogenesis by changing the fat distribution. BMC Gastroenterol. 2015;15:177. 26678309 10.1186/s12876-015-0409-9 PMC4683865

[22] Persico M, Masarone M, Damato A, et al. Non-alcoholic fatty liver disease and eNOS dysfunction in humans. BMC Gastroenterol. 2017;17:35. 28264657 10.1186/s12876-017-0592-y PMC5340006

[23] Kumar KG, Trevaskis JL, Lam DD, Sutton GM, Koza RA, Chouljenko VN, et al. Identification of adropin as a secreted factor linking dietary macronutrient intake with energy homeostasis and lipid metabolism. Cell Metab. 2008;8(6):468–81. 19041763 10.1016/j.cmet.2008.10.011 PMC2746325

[24] Kutlu O, Altun Ö, Dikker O, Aktaş Ş, Özsoy N, Arman Y, Özgün Çil E, Özcan M, AydınYoldemir Ş, Akarsu M, Toprak İD, Kırna K, Kutlu Y, Toprak Z, Eruzun H, Tükek T. Serum Adropin Levels Are Reduced in Adult Patients with Nonalcoholic Fatty Liver Disease. Med PrincPract. 2019;28(5):463–9. 10.1159/000500106 PMC6771072 30995640

[25] Yilmaz M, Odabas O, Karaaslan M, Guler OF, Toprak T, Bicer S, Tonyali S. Predicting risk of erectile dysfunction in patients with nonalcoholic fatty liver disease. Andrologia. 2021;53(7):e14091. 33951744 10.1111/and.14091

[26] Duman DG, Biçakci E, Çelikel ÇA, Akbal C. Nonalcoholic Fatty Liver Disease is Associated with Erectile Dysfunction: A Prospective Pilot Study. J Sex Med. 2016;13(3):383–8. 26853046 10.1016/j.jsxm.2015.12.030

[27] World Medical Association. World Medical Association Declaration of Helsinki: Ethical principles for medical research involving human subjects. JAMA. 2013;310(20):2191–4. 24141714 10.1001/jama.2013.281053

[28] Ferraioli G, Monteiro LBS. Ultrasound-based techniques for the diagnosis of liver steatosis. World J Gastroenterol. 2019;25(40):6053. 31686762 10.3748/wjg.v25.i40.6053 PMC6824276

[29] Eslam M, Newsome PN, Sarin SK, Anstee QM, Targher G, Romero-Gomez M, et al. A new definition for metabolic dysfunction-associated fatty liver disease: An international expert consensus statement. J Hepatol. 2020;73(1): 202–209. 10.1016/j.jhep.2020.03.039 32278004

[30] Shamloul R, Ghanem H, Abou-Zeid A. Validity of the Arabic version of the sexual health inventory for men among Egyptians. Int J Impot Res. 2004;16(5):452–5. 15175638 10.1038/sj.ijir.3901248

[31] Spitzer RL, Kroenke K, Williams JB, et al. A brief measure for assessing generalized anxiety disorder: the GAD-7. Arch Intern Med. 2006;166(10):1092–7. 16717171 10.1001/archinte.166.10.1092

[32] Sawaya H, Atoui M, Hamadeh A, Zeinoun P, Nahas Z. Adaptation and initial validation of the Patient Health Questionnaire–9 (PHQ-9) and the Generalized Anxiety Disorder–7 Questionnaire (GAD-7) in an Arabic speaking Lebanese psychiatric outpatient sample. Psychiatry Res. 2016;30(239):245–52. 10.1016/j.psychres.2016.03.030 27031595

[33] Kroenke K, Spitzer RL, Williams JB. The PHQ-9: validity of a brief depression severity measure. J Gen Intern Med. 2001;16(9):606–13. 11556941 10.1046/j.1525-1497.2001.016009606.x PMC1495268

[34] Kawaguchi T, Tsutsumi T, Nakano D, Torimura T. MAFLD: Renovation of clinical practice and disease awareness of fatty liver. Hepatol Res. 2022;52(5):422–32. 34472683 10.1111/hepr.13706

[35] Cobbina E, Akhlaghi F. Non-alcoholicfatty liver disease (NAFLD)–pathogenesis, classification, and effect on drug metabolizing enzymes and transporters. Drug Metab Rev. 2017;49(2):197–211. 28303724 10.1080/03602532.2017.1293683 PMC5576152

[36] Young S, Tariq R, Provenza J, Satapathy SK, Faisal K, Choudhry A, Friedman SL, Singal AK. Prevalence and profile of nonalcoholic fatty liver disease in lean adults: systematic review and Meta-Analysis. Hepatology communications. 2020;4(7):953–72. 32626829 10.1002/hep4.1519 PMC7327210

[37] Burnett AL, Lowenstein CJ, Bredt DS, et al. Nitric oxide: A physiologic mediator of penile erection. Science. 1992;257:401–3. 1378650 10.1126/science.1378650

[38] Burnett AL. The role of nitric oxide in erectile dysfunction: implications for medical therapy. J ClinHypertens (Greenwich). 2006;8(12 Suppl 4):53–62. 10.1111/j.1524-6175.2006.06026.x PMC8109295 17170606

[39] Kuloglu T, Aydin S. Immunohistochemical expressions of adropin and inducible nitric oxide synthase in renal tissues of rats with streptozotocin-induced experimental diabetes. Biotech Histochem. 2014;89:104–10. 23957703 10.3109/10520295.2013.821713

[40] Topuz M, Celik A, Aslantas T, Demir AK, Aydin S, Aydin S. Plasma adropin levels predict endothelial dysfunction like flow-mediated dilatation in patients with type 2 diabetes mellitus. J Investig Med. 2013;61:1161–4. 24113736 10.2310/JIM.0000000000000003

[41] Osuna JA, Gómez-Pérez R, Arata-Bellabarba G, et al. Relationship between BMI, total testosterone, sex hormone binding-globulin, leptin, insulin and insulin resistance in obese men. Arch Androl. 2006;52:355–61. 16873135 10.1080/01485010600692017

[42] Eguchi Y, Mizuta T, Sumida Y, et al. The pathological role of visceral fat accumulation in steatosis, inflammation, and progression of nonalcoholic fatty liver disease. J Gastroenterol. 2011;46(Suppl 1):70–8. 21042922 10.1007/s00535-010-0340-3

[43] Herman AP, Krawczynska A, Bochenek J, et al. LPS-induced inflammation potentiates the IL-1beta-mediated reduction of LH secretion from the anterior pituitary explants. Clin Dev Immunol. 2013;2013:926937. 23956762 10.1155/2013/926937 PMC3730224

[44] Crocetto F, Barone B, Manfredi C, Trama F, Romano L, Romeo M, Russo G, Sicignano E, Persico F, Aveta A, Spirito L, Napolitano L, Imbimbo C, Tarantino G. Are insulin resistance and non-alcoholic fatty liver disease associated with Peyronie's disease? A pilot study. J PhysiolPharmacol. 2022;73(1). 10.26402/jpp.2022.1.05. PMID: 35639037. 10.26402/jpp.2022.1.05 35639037

[45] Defeudis G, Mazzilli R, Gianfrilli D, Lenzi A, Isidori AM. The CATCH checklist to investigate adult-onset hypogonadism. Andrology. 2018;6(5):665–79. 29888533 10.1111/andr.12506

[46] Ragab A, Ahmed MH, Reda Sayed A, EldinAbdelbary DAK, GamalEl Din SF. Serum nesfatin-1 level in men with diabetes and erectile dysfunction correlates with generalized anxiety disorder-7: A prospective comparative study. Andrology. 2023;11:307–15. 35871269 10.1111/andr.13237

[47] Targher G, Chonchol MB, Byrne CD. CKD and non-alcoholic fatty liver disease. AJKD. 2014;64:638–52. 25085644 10.1053/j.ajkd.2014.05.019

[48] Ix JH, Sharma K. Mechanisms linking obesity, chronic kidney disease, and fatty liver disease: The roles of fetuin-A, adiponectin, and AMPK. J Am Soc Nephrol. 2010;21:406–12. 20150538 10.1681/ASN.2009080820 PMC4473254

[49] Marcuccilli M, Chonchol M. NAFLD and Chronic Kidney Disease. Int J Mol Sci. 2016;17(4):562. 27089331 10.3390/ijms17040562 PMC4849018

[50] Sun DQ, Ye FZ, Kani HT, Yang JR, Zheng KI, Zhang HY, et al. Higher liver stiffness scores are associated with early kidney dysfunction in patients with histologically proven non-cirrhotic NAFLD. Diabetes Metab 2020;46(4):288–295. 10.1016/j.diabet.2019.11.003 31786360

[51] Jones RWA, Rees RW, Minhas S, et al. Oxygen free radicals and the penis. Expert OpinPharmacother. 2002;3(7):889–97. 10.1517/14656566.3.7.889 12083989

[52] Cirakogl A, Yuce A, Benli E, Arici YK, Dugeroglu H, Ogreden E. Is erectile dysfunction an early clinical symptom of chronic kidney disease? Aging Male. 2021;24:24–8. 34096824 10.1080/13685538.2021.1936483

[53] Hasanain AFA, Mahdy RE, Mahran AMA, Safwat ASM, Mohamed AO, Abdel-Aal SM. Erectile dysfunction in patients with nonalcoholic fatty liver disease. Arab J Gastroenterol. 2017;18(1):21–4. 28325476 10.1016/j.ajg.2017.02.002

[54] Ali MK, Chwastiak L, Poongothai S, et al.; Independent Study Group. Effect of a collaborative care model on depressive symptoms and glycated hemoglobin, blood pressure, and serum cholesterol among patients with depression and diabetes in India: the INDEPENDENT randomized clinical trial. JAMA. 2020;324(7):651–662. 10.1001/jama.2020.11747 PMC7435347 32809002

